# Supplementary material for: Functional bottlenecks can emerge from non-epistatic underlying traits
Source: PLoS Comput Biol. 2026 Mar 16;22(3):e1014000. doi: 10.1371/journal.pcbi.1014000 (PMC13020760; doi:10.1371/journal.pcbi.1014000)
Supplement: S1 Text — (PDF) [file pcbi.1014000.s001.pdf]

Supplementary Material for
"Functional bottlenecks can emerge from non-epistatic underlying traits"

Anna Ottavia Schulte,[1] Samar Alqatari,[2] Saverio Rossi,[1] and Francesco Zamponi[1]

[1] *Dipartimento di Fisica, Sapienza Università di Roma, Piazzale Aldo Moro 5, 00185 Rome, Italy*
[2] *Department of Physics and The James Franck and Enrico Fermi Institutes, The University of Chicago, Chicago, IL 60637, USA*

(Dated: February 13, 2026)

S1. DETAILS ON THE ANALYSIS OF EXPERIMENTAL DATA

A. Data collection

The experimental measurements that were analyzed in the main text were obtained from the public repository associated with Ref. [1]. These are raw sequencing data capturing the genotype variants present in the red and blue channel after two-color Fluorescence Activated Cell Sorting. Following the procedure detailed in Ref. [1] (see the original paper for details), sequences with low quality scores and noisy measurements were filtered out. The enrichment scores for each genotype $\mathbf{a}$ were computed counting the occurrence of each variant in the two channels normalized to its abundance in the input channel, i.e. before sorting. We further normalized the enrichment scores with respect to the ones of the reference variants, such that $F_R(\mathbf{a}_R) = F_B(\mathbf{a}_B) = 1$, and afterwards removed global epistasis via a non-linear function

$$E_f = \phi^{-1}(F_f) = F_f^{0.44} \ , \tag{S1}$$

as proposed by the authors of Ref. [1], separately for the blue ($f = B$) and red ($f = R$) fluorescence.

B. Results with non-normalized enrichment scores

In the main text, and as discussed in Sec. S1 A, we normalized the enrichment scores to the reference values, a choice that was not made in the original Ref. [1]. Using normalized enrichment scores, we find that no path of single mutations exists that connects the two reference variants while always maintaining $|E(\mathbf{a})| \geq E_{\max}^{\mathrm{ref}}$, hence we need to consider $E_{\mathrm{th}} < E_{\max}^{\mathrm{ref}}$ in order to preserve the connection between the reference variants, as discussed in the main text. Instead, Ref. [1] shows a topology of the space of paths connecting genotypes with $|E(\mathbf{a})| \geq E_{\mathrm{th}}$ and $E_{\mathrm{th}} = E_{\min}^{\mathrm{ref}}$. This discrepancy arises entirely from the choice of not normalizing the phenotypic trait.

The space of paths presented in Ref. [1] is constructed by defining a phenotype $E = \sqrt{\epsilon_B^2 + \epsilon_R^2}$, where ϵ_B and ϵ_R are the enrichment scores in the two channels, which are *not* normalized with respect to the enrichment scores of the red and blue reference variants. However, they are rescaled to reflect the experimental brightness ratio between the parental blue and red genotypes, which is 0.172, and subsequently transformed using the non-linear function in Eq. S1 to remove global epistasis. To carry out a proper comparison with our analysis, we reproduce the space of paths of Ref. [1] modifying their definition of phenotype to $\widetilde{E} = \epsilon_B - \epsilon_R$. Fig. Aa shows that this is a minor change: because $\epsilon_R \sim 0$ when $\epsilon_B > 0$ and vice versa, the two phenotypes are essentially exclusive and as a consequence $E = \sqrt{\epsilon_B^2 + \epsilon_R^2} \sim \max(\epsilon_B, \epsilon_R) \sim |\widetilde{E}|$. Hence, $\widetilde{E}$ is numerically very close to the score used in the original Ref. [1], while keeping the negative sign for the red phenotype.

Compared to the normalized case shown in the main text, the resulting distribution of phenotypes, shown in Fig. Ab, is not symmetric around zero: the red reference sequence has $|\widetilde{E}_R^{\mathrm{ref}}| = 0.56$ and the blue one is more than twice as large, with $\widetilde{E}_B^{\mathrm{ref}} = 1.49$. This asymmetry arises entirely from the absence of normalization. When looking for the largest value of the functionality threshold $\widetilde{E}_{\mathrm{th}}$ such that at least one viable mutational path exists between the two reference variants, we find that $\widetilde{E}_C$ saturates to $\widetilde{E}_{\min}^{\mathrm{ref}}$ and does not produce a topology with a single jump. The topology is the same as the one shown in Ref. [1], and we consistently find that $\widetilde{E}_C = \widetilde{E}_{\min}^{\mathrm{ref}}$ is the critical value above which any connection between the two reference sequences is lost. This is exactly what we would expect given the imbalance between the two reference phenotypes. However, given this imbalance, we believe that choosing a symmetric threshold, i.e. choosing the same $\widetilde{E}_{\mathrm{th}}$ for both functions, is not appropriate.

We therefore believe that the choice of normalizing the enrichment scores to the reference variants provides a better view of the global fitness landscape, with the distribution of phenotypic values centered around zero. This choice results in a critical threshold $\widetilde{E}_C < \widetilde{E}_{\mathrm{ref}}$, as discussed in the main text.

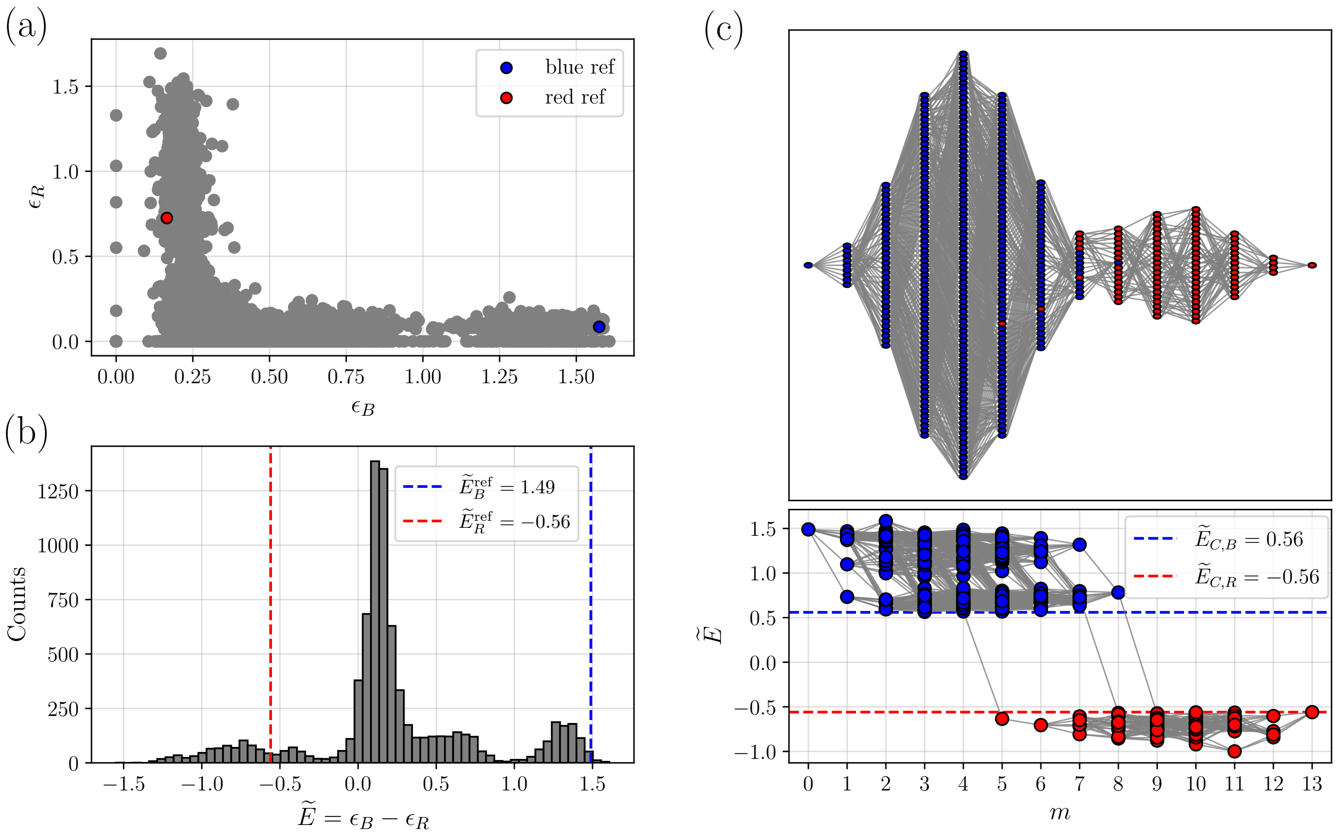

Fig A. (a) Scatter plot of the enrichment scores in the blue and the red channel, ϵ_B and ϵ_R, not normalized with respect to the reference values. For the blue reference variant $\epsilon_B = 1.58$, $\epsilon_R = 0.09$. For the red one $\epsilon_B = 0.17$, $\epsilon_R = 0.73$. (b) Histogram of the values of $\widetilde{E}$ obtained for each of the 2^{13} variants. The red and blue lines correspond respectively to the reference values $\widetilde{E}_R^{\rm ref}$ and $\widetilde{E}_B^{\rm ref}$. (c) Topology of the space of paths obtained keeping only genotypes $\mathbf{a}$ with $|\widetilde{E}(\mathbf{a})| > \widetilde{E}_C$, where $\widetilde{E}_C = 0.56$ (dashed lines) is the largest possible value such that the red and blue reference variants remain connected. The lower panel reports the values of $\widetilde{E}(\mathbf{a})$ for each functional genotype (blue dots for $\widetilde{E} > \widetilde{E}_C$ and red dots for $\widetilde{E} < -\widetilde{E}_C$) as a function of the number of mutations m from the blue reference sequence, with the gray lines connecting pairs of genotypes that differ by a single mutation. The upper panel shows the resulting graph of connections.

C. Removing network epistasis from the data

From now on, we stick to the choice made in the main text, of normalizing the enrichment scores to the ones of the reference variants. We note that even after the non-linear fitness function has been inverted by the fitting procedure we described, the effect of epistasis is still present in the data. This is due to the fact that the fitness cannot be simply expressed as a nonlinear function of an additive trait. An interesting test is then to try and remove this second source of epistasis (network epistasis) and check whether one can still obtain a topology similar to the one discussed in Refs. [1, 2] and in the main text. We do so by defining an additive phenotype $E_f^{(1)}$ obtained expanding the phenotypes E_f around their respective reference variant $\mathbf{a}_f$ and cutting the higher order terms, namely

$$E_f^{(1)}(\mathbf{a}) = E_f(\mathbf{a}_f) + \sum_{i=1}^{13} h_i^{f,(1)}(a_{f,i} - a_i) \ . \tag{S2}$$

After linearization, we combine the two phenotypes into a single additive phenotype $E^{(1)}(\mathbf{a}) = E_B^{(1)}(\mathbf{a}) - E_R^{(1)}(\mathbf{a})$.

Fig. Ba shows a scatter plot of the approximated additive phenotype $E^{(1)}(\mathbf{a})$ against $E(\mathbf{a})$. In the figure we highlight the reference sequences (blue and red stars) along with the ones that are at a small Hamming distance from the blue (d_B) and red (d_R) variants. One notices that E and $E^{(1)}$ are not simply linearly related, even if they have a relatively high Spearman correlation coefficient of 0.7. This suggests that the first order expansion is not able to fully capture all mutational effects and that network epistasis is indeed present in the data [1]. We note that the variants at small

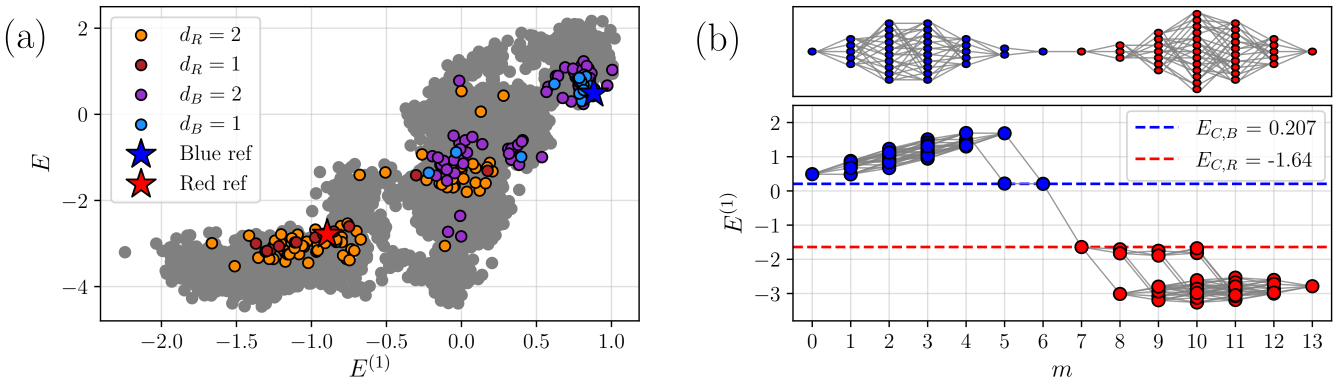

Fig B. Linearization of the phenotypes according to the expansion described in Eq. (S2). (a) Scatter plot of the phenotype values shown in the main text, E, and after the expansion, $E^{(1)}$. The Spearman correlation coefficient is about 0.7. The red and blue stars correspond respectively to the red and blue reference variants. (b) Topology of paths surviving at the connectivity thresholds E_C^B and E_C^R. Here $E^{(1)}$ is the linearized additive phenotype.

Hamming distance from the reference ones display a better correlation than the very distant ones, as expected.

Keeping in mind that the linearization is only an approximation, we analyzed the space of viable paths connecting the two reference variants. Following the same approach described in the main text, we look for the largest value of E_{th} for which at least one path allowing for a functionality switch survives, and call this value E_C. However, because the two reference sequences in this case turn out to be quite asymmetric, with $E_B^{\text{ref}} \sim 0.49$ and $E_R^{\text{ref}} \sim 2.8$, we allowed for an asymmetric connectivity threshold [2] for the paths joining the two variants (see also the discussion in Sec. S1 B). Instead of increasing E_{th} symmetrically from 0 and impose $|E| > E_{\text{th}}$ for every path, we impose $E > E_{\text{th}}^B$ and $E < -E_{\text{th}}^R$ separately for the two phenotypes. The largest values for which a path survives are $E_C^B = 0.21$ and $E_C^R = 1.64$, and we obtain the topology shown in Fig. Bb. Note that in the end, $E_C^B/E_B^{\text{ref}} \sim 0.42$ and $E_C^R/E_R^{\text{ref}} \sim 0.59$, hence we always end up with a connectivity threshold at about half the reference phenotype. We observe a significant reduction in functional variants, approximately at half distance between the two reference sequences, and the emergence of a bottleneck topology.

S2. ANALYSIS OF ADDITIONAL DATA FOR SINGLE-MUTATION EFFECTS

In Ref. [3] an extensive review of combinatorial experiments similar to that of Ref. [1] is presented. The authors analyzed the results from ten studies (see references in [3]) of combinatorially complete fitness landscapes of seven different enzymes, totaling 1440 genotype-phenotype data-points. The experimental results for fitness are then trans-

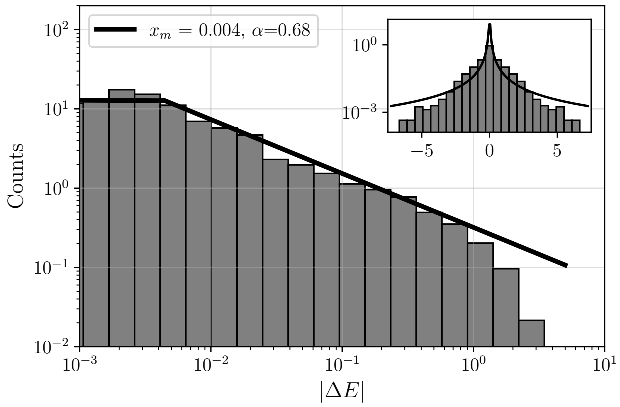

Fig C. Histogram of the absolute value $|\Delta E|$ for all SMEs extracted from the dataset of Ref. [3].

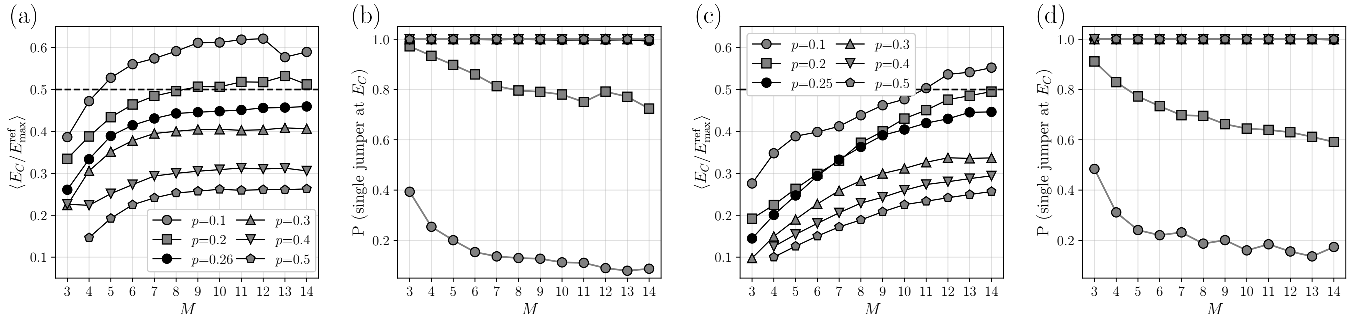

Fig D. Statistical properties of the space of paths upon varying the number of mutations M, at fixed values of p, for the Gaussian (a),(b), and the Pareto cutoff (c),(d) distributions. (a),(c) The average value of $E_C/E^{\rm ref}_{\rm max}$. (b),(d) The fraction of single jumpers present at E_C.

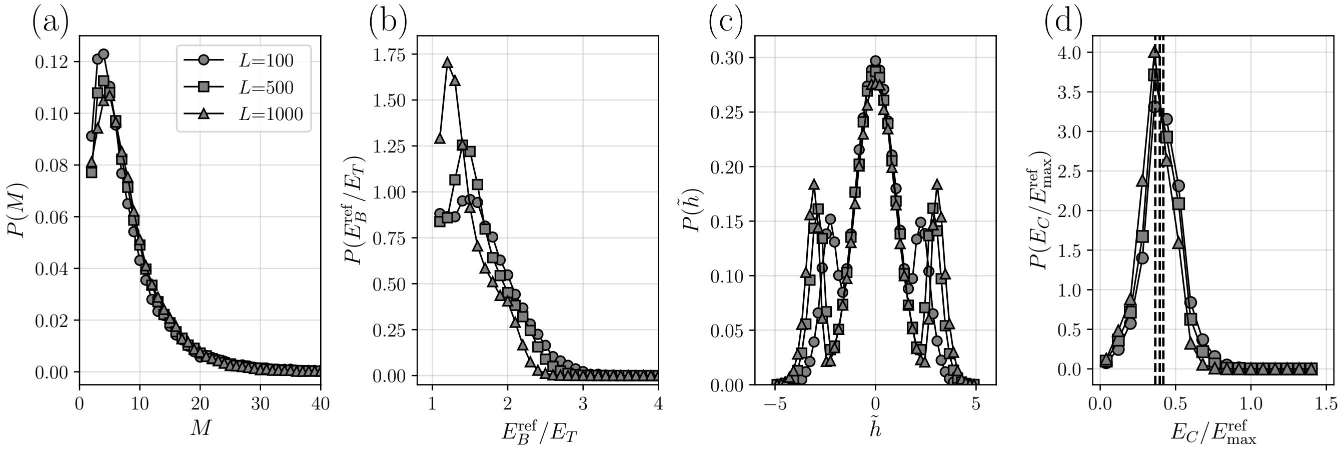

Fig E. Statistics of a few relevant quantities for the model with Gaussian distribution, for $L = 100, 500, 1000$. (a) Distribution of the number of mutations. (b) Distribution of the value of the positive (blue) reference phenotype divided by E_T. (c) Distribution of the selected SMEs $\tilde{h}$. (d) Distribution of $E_C/E^{\rm ref}_{\rm max}$.

lated into an underlying phenotype by attempting at removing non-linearity (see Ref. [3] for details), and the authors provide the resulting distribution of SMEs that can be downloaded from the paper repository. It should be noted, however, that most of these landscapes connect a reference wild type to an evolved variant with increased fitness, rather than two variants with distinct functionality.

In Fig. C, we show the same distribution as in Ref. [3], plotted similarly to the other figures in this paper. Once again the fit with the Pareto distribution seems to perform well, although with different values of x_m and α. Notice that the uniform behavior at small $|\Delta E|$, as well as the cutoff at large $|\Delta E|$, are not as sharp as in the data from Ref. [1]. This is probably due to the fact that Ref. [3] combined results for many different experiments, which have different sensitivity and a different range of measurable fitness.

S3. ADDITIONAL RESULTS ON THE STYLIZED MODEL

A. Evolution of the space of paths with the number of mutations

In the main text we presented the statistics of $E_C/E^{\rm ref}_{\rm max}$ for the calibrated models, i.e. for the parameter choices p and E_T that maximize both the connectivity threshold $E_C/E^{\rm ref}_{\rm max}$ and the probability of observing bottleneck structures. These two quantities are shown in Fig. D as a function of the number of mutations M separating the reference variants, for different values of p and E_T. As explained in the main text, the dotted line at $E_C = 0.5E^{\rm ref}_{\rm max}$ corresponds to the maximum value of connectivity threshold that can be found when observing a bottleneck with a single functionality switch. At large p, it can be seen that although the fraction of bottlenecks remains equal to

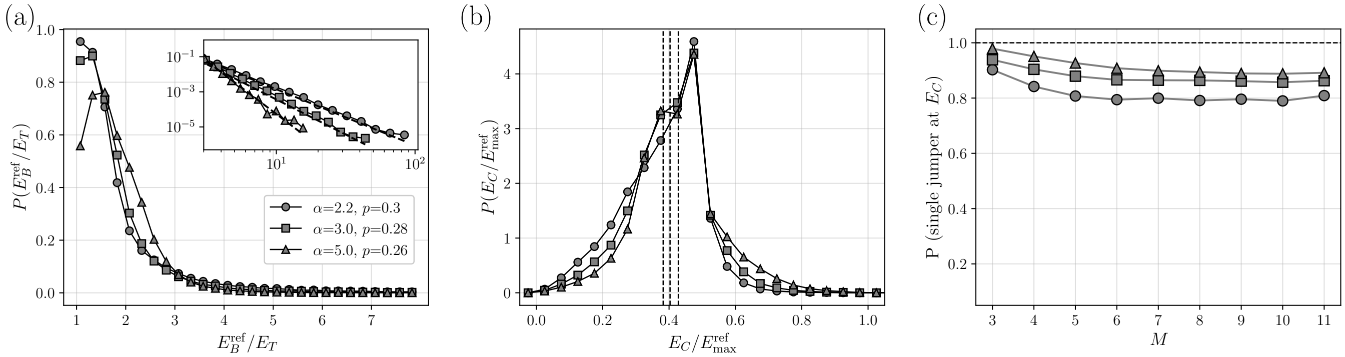

Fig F. Distribution of the relevant quantities for the model with Pareto distributed h values. Results are shown for different exponents $\alpha = 2.2$, 3.0, and 5.0. (a) Distribution of $E_B^{\rm ref}/E_T$. Inset: tails of the distributions in log-log scale. The dashed lines are obtained fitting with an exponent equal to α. (b) Distribution of the value of $E_C/E_{\rm max}^{\rm ref}$. (c) Fraction of single jumpers at E_C as a function of the number of mutations M.

one, $\langle E_C/E_{\rm max}^{\rm ref}\rangle$ decreases with increasing p. Low values of $E_C/E_{\rm max}^{\rm ref}$ correspond to a connectivity threshold that lies deep within the region of non-functionality, making the corresponding evolutionary trajectories unlikely to occur. On the other hand, for small p, corresponding to a random selection of mutational effects, the fraction of observed bottlenecks decreases significantly.

For the values of p and E_T chosen after calibration (black dots), we observe simular results to Ref. [2]. The probability of a bottleneck remains always equal to one, while the value of $E_C/E_{\rm max}^{\rm ref}$ increases with M and saturates to the expected maximum.

B. Dependence on system size

All the results reported in the main text have been obtained by fixing the number of SMEs to $L = 500$. This would correspond to a protein of 500 amino acids or to an elastic network with 500 edges. To check the consistency of our results we also perform simulations varying the length L of the binary sequence. In particular we study $L = 100$ and $L = 1000$ as well. We focus on the same parameters used in the main text, namely a Gaussian with $\mu = 0$, $\sigma = 1.0$ and we determine p, E_T fixing $\langle M\rangle \approx 8$. As expected, the required value of E_T increases with L, because the maximum of the h_i increases proportionally to $\sqrt{\log L}$ as dictated by extreme value statistics. The growth is slow enough, however, and it is compensated by the tuning of E_T, in such a way that the results discussed in the main text are not significantly affected by the choice of the system size. In Fig. E, panels (a) and (b), we plot the distribution of the number of mutations and of the value of $E_B^{\rm ref}/E_T$, respectively. We see that, with an appropriate choice of E_T and p for each L, the results are almost indistinguishable for the three values of L. In panels (c) we plot the distribution of the values of h_i selected by the tuning procedure. We see that, as L increases, the peak of strongly beneficial (or strongly deleterious) mutations shifts towards larger absolute values, sharpening the separation between greedy and random steps. Finally, in panel (d) we show the distribution of $E_C/E_{\rm max}^{\rm ref}$, which is once again unaffected by the choice of L.

C. Heavy tailed distribution of single-mutation effects

The analysis of experimental data for SMEs suggests that the initial part of the distribution is described by a heavy-tailed Pareto distribution, which is then cutoff at large $|\Delta E|$, in part because of biological reasons (the effect of a mutation on fitness cannot be too large), in part due to the limited range of fitness variations that can be measured in experiments.

Yet, it is interesting to check what would happen in our model if the heavy tail would be allowed to continue up to infinity (keeping in mind that the finite value of L will induce a cutoff anyways). Hence, we show here some results obtained for various choices of the input distribution $P(h)$ with Pareto tails decaying according to an exponent α as in Eq.(5) of the main text, without cutoff. We analyze different values of $\alpha = 2.2, 3, 5$, for which the variance of the distribution is finite. We fix the system size to $L = 500$ as in the main text, to facilitate comparison. We then proceed as described in the main text and we characterize the statistics of $E_f^{\rm ref}/E_T$, $E_C/E_{\rm max}^{\rm ref}$, and the topologies that arise

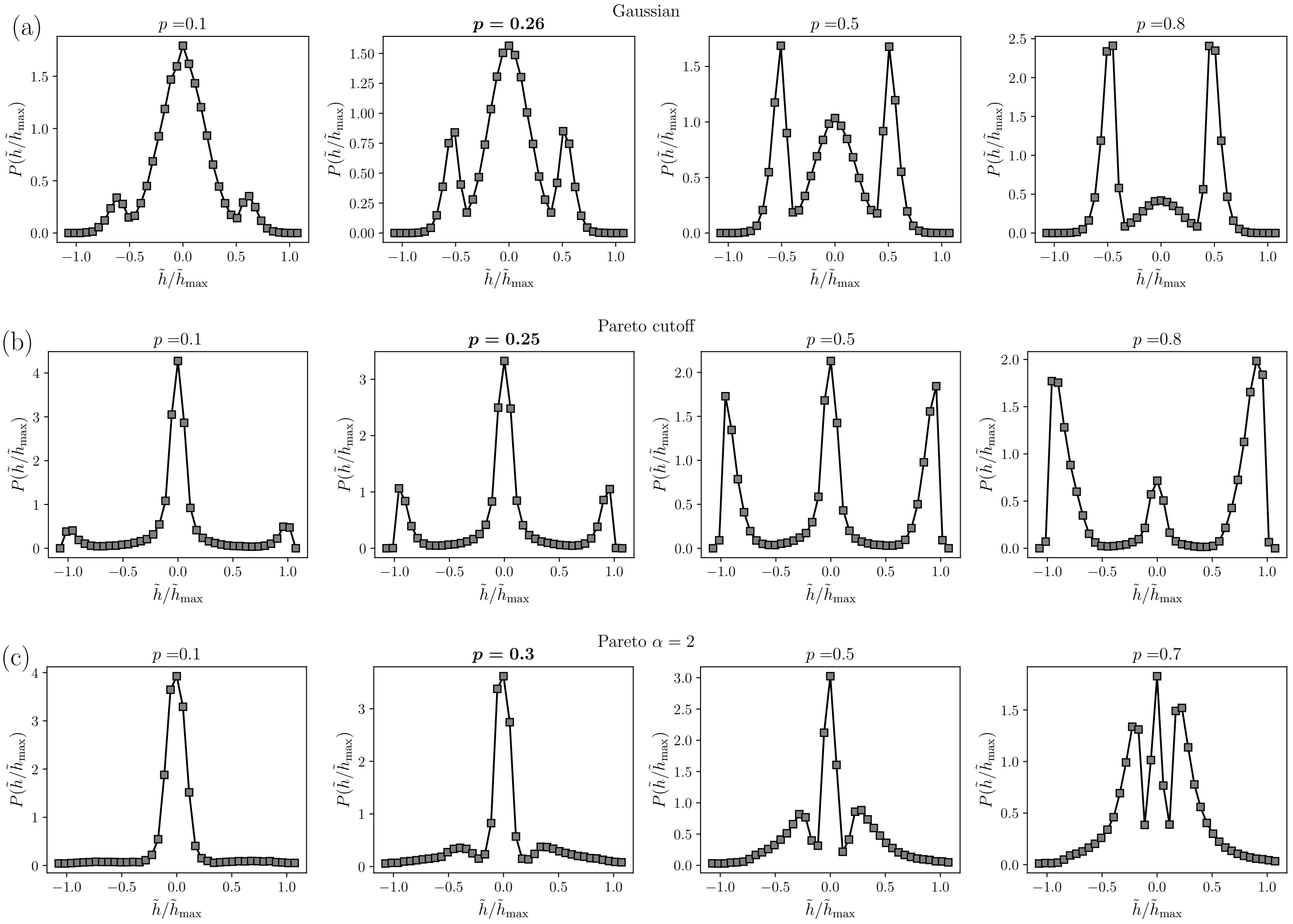

Fig G. Distribution of $P(\tilde{h}/\tilde{h}_{\max})$. Comparison between calibrated (bold $\boldsymbol{p}$) and non calibrated models. (a) Gaussian input distribution $P(h)$. Calibrated model for $p = 0.26$, $E_T = 2.0$. (b) Pareto cutoff distribution $P(h)$: $\alpha = 0.7$, $x_m = 1$, $\epsilon = 2$. Calibrated model for $p = 0.25$, $E_T = 1.1$. (c) Pareto distribution $P(h)$ with $\alpha = 2.0$. Calibrated model for $p = 0.3$, $E_T = 8.0$.

from this input distribution of SMEs. The results are shown in Fig. F, for the optimal choice of p and E_T.

For this choice of the parameters we find $\langle E_C/E^{\text{ref}}_{\max}\rangle \simeq 0.5$, which is the largest possible value of E_C maximizing the fraction of bottleneck topologies in the ensemble. We note that if there is only one greedy step, the value of the reference phenotype E^{ref}_B is given by the sum of $M-1$ values drawn from $P(h)$ and a value drawn from $P^L_{\max}(h_{\max})$, the probability distribution of the maximum value of the L variables h, which is easily derived from extreme value statistics. If $P(h)$ has heavy tails, we expect the sum to be dominated by the maximum and, as a consequence, $P(E^{\text{ref}}_B) \sim P^L_{\max}(h_{\max})$. The same reasoning works for the red reference phenotype E^{ref}_R. In Fig. Fa we show the plot of E^{ref}_B for different values of the exponent α. In the inset of this panel one can see how the tails of such distribution decay following a power law with the same exponent α, as expected.

The distribution of $E_C/E^{\text{ref}}_{\max}$, shown in Fig. Fb, seems to be rather independent of the exponent α, and is centered around 0.5 as observed in the previous cases. What is different is the fraction of single jumpers at E_C, shown in Fig. Fc. At variance with the Gaussian and Pareto cutoff cases, here the fraction of bottleneck topologies is different from one even for the optimal values of p and E_T. This difference is due to the fat tails distribution of the reference phenotype E^{ref}_f. Since the distribution is very broad, it is possible to have a very unbalanced situation in which one of the two reference phenotypes is much larger than the other (in particular, when $E^{\text{ref}}_{\max} \gtrsim 2E^{\text{ref}}_{\min}$), which allows for the saturation of E_C to $E^{\text{ref}}_{\min}$ and the absence of a bottleneck. As expected, this effect disappears for larger values of α, when the tails are less pronounced, and absent in the Gaussian and Pareto cutoff distributions.

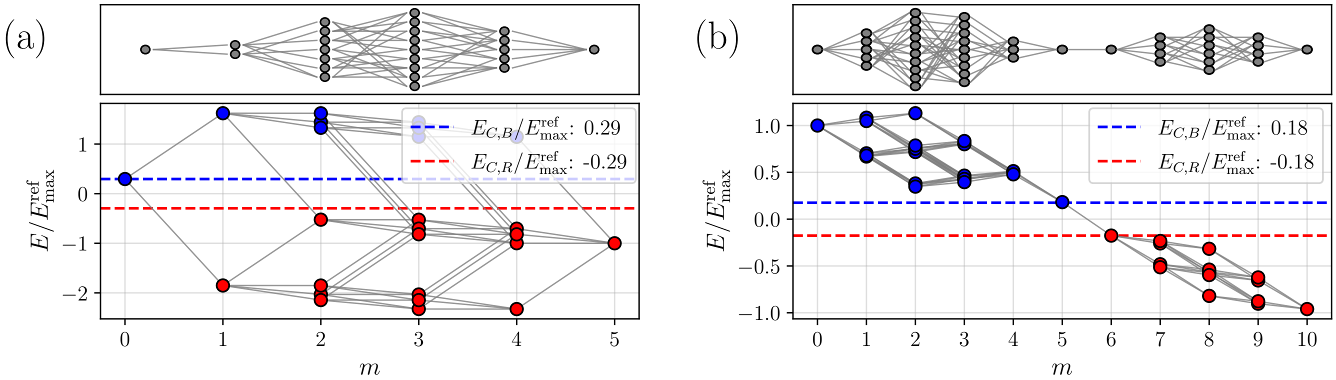

Fig H. Topology of paths surviving at the connectivity threshold for two instances drawn from uncalibrated models. E and E_C are scaled with respect to the largest of the two reference genotypes $E^{\rm ref}_{\rm max}$. (a) Gaussian input distribution $P(h)$, $p = 0.1$, $E_T = 0.08$. Note the typical behaviour: the two reference genotypes are unbalanced, $E^{\rm ref}_B/E_T = 2.73$, $E^{\rm ref}_R/E_T = -9.25$, $E^{\rm ref}_B/E^{\rm ref}_R = 3.39$; the connectivity threshold reaches its upper bound, which is the smallest between the two reference genotypes $E^{\rm ref}_{\rm min}$. (b) Gaussian input distribution $P(h)$, $p = 0.8$, $E_T = 8.2$. Here the reference genotypes are symmetric and close to E_T: $E^{\rm ref}_B/E_T = 1.11$, $E^{\rm ref}_R/E_T = -1.06$, $E^{\rm ref}_B/E^{\rm ref}_R = 1.04$; there is a single jumper mutation at half distance but the connectivity threshold is very small.

D. Balance between neutral and strongly non-neutral mutations

A central finding of our work is that the emergence of functional bottlenecks is contingent upon a specific balance within the distribution of *a posteriori* 'fixed' mutational effects, $P(\tilde{h})$. While the *a priori* distribution of available mutations, $P(h)$, can be relatively flexible, the realized mutational neighborhood of the reference genotypes must exhibit a high degree of heterogeneity to produce the desired topology. In Fig. G, we illustrate this balance by comparing calibrated models against non-calibrated ones. The calibration procedure essentially ensures that the two reference genotypes are separated by an underlying trait distance that allows for a 'neutral bulk' of mutations punctuated by a sufficient frequency of large-effect mutations. As illustrated in Fig. G, when the model is not properly calibrated, the resulting distribution $P(\tilde{h})$ fails to achieve this critical composition in two distinct ways. If the fraction of large-effect mutations is too small, the fitness landscape remains overly smooth and accessible, preventing the formation of fitness valleys (at too small p). Conversely, if too many large-effect mutations dominate the distribution (at too large p), the landscape becomes fragmented, and the selection pressure required to form a 'corridor' between functional states must be too small. These results substantiate our claim that functional bottlenecks do not emerge by default from any nonlinear mapping. Rather, they require a specific balance between a majority of nearly neutral mutations and a minority of strongly non-neutral ones, which is provided by our procedure to construct the reference genotypes. This provides a clear example of why the construction is non-trivial: the bottleneck structure is a direct consequence of the heterogeneity in mutational effect sizes, and it vanishes when this heterogeneity is removed or incorrectly tuned.

In Fig. H we show typical examples of paths that emerge when the model is not properly calibrated. As already stated before, if the fraction of large-effect mutations is too small, no single jumper mutation stands out and the functionality switch can occur at any step along the evolutionary path. This is illustrated in Fig. Ha: the fitness landscape is smooth and no bottleneck emerges during evolution. Conversely, if large-effect mutations dominate the distribution, as in Fig. Hb, the jump required to switch functionality is small: evolution has to accommodate substantial functionality loss with respect to the reference genotypes before being able to switch functionality, thus making these paths very unlikely to occur.

[1] F. J. Poelwijk, M. Socolich, and R. Ranganathan, Learning the pattern of epistasis linking genotype and phenotype in a protein, Nature communications **10**, 4213 (2019).
[2] S. Alqatari and S. R. Nagel, Evolutionary pathways in epistatic mechanical networks, Proceedings of the National Academy of Sciences **123**, e2505183123 (2026).
[3] K. Buda, C. M. Miton, and N. Tokuriki, Pervasive epistasis exposes intramolecular networks in adaptive enzyme evolution, Nature Communications **14**, 8508 (2023).
