# Supplementary material for: Functional bottlenecks can emerge from non-epistatic underlying traits
Source: PLoS Comput Biol. 2026 Mar 16;22(3):e1014000. doi: 10.1371/journal.pcbi.1014000 (PMC13020760; doi:10.1371/journal.pcbi.1014000)
Supplement: Attachment — Submitted filename: resub_cover_letter.pdf [file pcbi.1014000.s002.pdf]

Additional Editor Comments:

The manuscript has been reviewed by two senior experts in the field. While their overall enthusiasm on this study differs, they converge on a series of points to address, regarding both the main model assumptions and the comparison with key recent literature. Both reviewers agree that the manuscript addresses an interesting and timely question.

My own view aligns more with reviewer 1, and therefore I will be happy to reconsider the manuscript once all reviewers' remarks have been fully addressed. Note however that additional work on the model and a major revision on the manuscript addressing central claims, relation with literature, and model-data comparisons are required for this revision.

Kind Regards
MCL

We warmly thank the editor for organizing a careful review process and for the positive assessment. We appreciate the constructive feedback from both referees and have addressed all comments in this reply and modified the manuscript accordingly. We believe these revisions have strengthened the manuscript.

Should additional review be helpful, we respectfully suggest Prof. Joseph Thornton (University of Chicago) or Prof. John Barton (University of Pittsburgh) as potential referees with relevant expertise.

Best regards,
The authors

Reviewer #1:

The manuscript investigates to what extent a particular fitness landscape structure, where two distinct phenotypic functions are connected in genotype space through a functional bottleneck consisting of a small number of genotypically accessible paths, can arise purely from global epistasis. The latter term refers to a scenario where a non-epistatic (i.e., linear/additive) genotype-phenotype map is combined with a nonlinear phenotype-fitness map to yield an epistatic (and possibly rugged/complex) genotype-fitness landscape. The broader context of the work is the general question as to whether empirically observed fitness landscape structures can be generated by global epistasis without invoking additional "network" epistasis on the level of the genotype-phenotype map. The authors provide a clear introduction to the context of the work and answer the guiding research question affirmatively by constructing an explicit global epistasis model that produces the desired landscape structure. The paper is clearly written and well organized, and makes an important contribution to our understanding of protein evolution. I recommend publication in PLoS Computational Biology after the following comments have been addressed.

1. My main concern about the manuscript is that it did not become clear to me how surprised I should be by the result. The construction of the toy model involves a lot of deliberate fine-tuning towards the desired bottleneck structure, and it is hard to see how this procedure could have NOT worked. More specifically, at the end of the first paragraph of the Discussion the authors write "When mutational effects are broadly distributed, evolutionary paths between distinct phenotypes are constrained, leading to the formation of narrow evolutionary corridors." which suggests that such corridors will NOT form if the effects are not sufficiently heterogeneous, but (as far as I could see) this claim is not substantiated by systematically varying the amount of effect size heterogeneity in the model. This central point needs to be clarified.

We thank the referee for raising this important point, and agree generally that whether the results are surprising or not depends on one's prior expectations. We make the following modifications to our manuscript so that, regardless of how surprising the results may be to the reader, more clearly demonstrate their non-triviality and relevance to the broader questions of epistasis and functional bottlenecks in evolutionary systems.

First, we clarify the distinction between:

- the *a priori* distribution of mutations, P(h), to be interpreted as the “pool” of mutations that can be chosen by evolution,
- and the *a posteriori* distribution after selection, P(h tilde), which represent the mutations “fixed” by evolution.

We show in our work that the former can be essentially arbitrary, while the latter needs to achieve a right balance between neutral and non-neutral mutations, i.e. be sufficiently heterogeneous. In our modeling approach, this required feature of P(h tilde) is induced by our procedure to construct the two reference genotypes, which is calibrated to produce the desired output.

As one can see in Fig. 4d, calibrated models exhibit a bulk of small values of h tilde, *i.e.*, neutral mutations, and a smaller, yet essential, amount of larger mutational effects. We added a more detailed discussion in the Supplementary Information, in particular a new figure S7, to show that when calibration is not properly done, we do not obtain the right balance of neutral vs non-neutral mutations: the fraction of large mutational effects is either too small or too large with respect to the bulk, and as a consequence we

do not obtain the bottleneck structures. This provides an example of what could go wrong, and why the construction is nontrivial.

We agree with the referee that the results may not be surprising from a statistical physics standpoint, but our analysis of biological data extends the claims to the biology literature where it is often claimed that network epistasis is needed for bottlenecks, which indicates that some part of the community has different beliefs. We thus think that our work can contribute positively to the broader debate.

2. In the discussion of the state of the art in the Introduction, I was surprised that the recent work from the lab of Andreas Wagner on large-scale high-throughput protein fitness landscapes is not mentioned. In particular, the investigation of the evolution of bacterial transcription factor binding sites towards three global E. coli transcription factors seems to consider an evolutionary scenario that is quite similar to the one addressed here (Westmann et al., https://doi.org/10.1101/2024.11.10.620926).

We thank the referee for suggesting this work, which we were not aware of. We carefully read the paper, but our feeling is that the landscapes presented in that work are quite different from the ones we consider. In fact, all the three combinatorial landscapes studied by Wagner et al. present no bottlenecks, and a significant degree of promiscuity of intermediates. We of course cited this work, which is certainly relevant to describe the state of the art, but we stressed that we focus on scenarios with bottlenecks. This work, with others, serves as an example of a landscape with no bottlenecks, which shows that of course bottlenecks are not always present. We mentioned this more explicitly in the introduction.

We should also note that the data are not publicly available and the authors of that paper did not respond to a request to share them, so we could not reanalyze those data.

3. At the end of the Introduction the authors claim that "the model is analytically solvable to some extent" and repeat this statement in the Discussion, but give no hints as to how such an analytic solution could be achieved. They should either provide more details or omit these statements.

We are sorry for these unsubstantiated claims. We have performed preliminary calculations that show that some quantities we computed numerically could also be computed analytically, such as the probability distribution of the two reference phenotypes. However, we did not carry on these computations to the end, so we did not include them in the paper. We removed the statement in the introduction and we better phrased the statement in the conclusions to specify that this is speculative.

4. Regarding the distinction of the present model from Fisher's geometric model (FGM) mentioned on page 6, I would like to point out that variants of FGM with multiple phenotypes have been considered previously in the literature (for example, G. Martin & T. Lenormand, Evolution 69 (2015) 1433–1447).

We thank the referee for pointing out this paper, and we added a citation to it.

Reviewer #2:

The work entitled “Functional bottlenecks can emerge from non-epistatic underlying traits” by Schulte et al. studies a mutant library data set to better understand epistasis and its effects on functional bottlenecks. The basic question, whether additive underlying trait which maps nonlinearly to fitness (global epistasis) is sufficient to produce functional bottlenecks or whether also interactions explicitly at the level of fitness are needed (network epistasis), is an interesting one. The manuscript tries to address

this question by analysing an existing data set of a mutant library (2^13 sequences) with corresponding fluorescent measurements in conjunction of a “toy” model.
Although the research question is of broad interest, the taken approach and the manuscript has substantial issues that make the work a poor match to PLOS CB.

We thank the referee for suggesting that the question we address is interesting. Below we try to better motivate our modeling choices and why we believe that they are relevant to the audience of PLOS CB.
We would like to stress, however - and we will reiterate this below - that the main point of our paper is the stylized model we introduce; the analysis of the existing set of mutants is only provided as a motivation, mostly for pedagogical purposes. We have stressed this more explicitly at the beginning of the “Results” section, to avoid misunderstandings.

1)The authors mix fitness with trait value “Note that here, fitness refers to fluorescence intensity, which is unrelated to reproductive success.” , possibly this is the norm also by others analysing comparable data but this problem seriously limits the interpretation of the results. In effect, the data allows to study the mapping between genotype and this trait, no more no less. This fundamental issue leads to difficulties later e.g. by needing to resort to arbitrariness of thresholds for what makes a viable genotype “As observed in Ref. [27] and evident from Fig. 1b, the threshold for defining a genotype as ‘functional’ is somewhat arbitrary, and playing with this threshold changes the topology substantially [38]. ”

As indicated by the referee, using some measurable trait as a proxy for fitness is a standard choice in this kind of experiment. Typically a measure of efficiency of some process (here fluorescence) is used. We note that similar choices have been made in the work by the group of A.Wagner pointed out by both referees, and that the data we used in our analysis come from a leading group in evolutionary biology and were published on Nature Communications. Hence, we do not believe that this is a limitation of our work.

Concerning the choice of threshold, this of course is arbitrary, not only in the modeling, but also fundamentally in biology: indeed, what makes a genotype viable depends on the selection pressure, which itself depends on the environment (either natural or laboratory). The fact that changing the selection pressure changes the topology is a fact, both in our model and in nature. The existence of a bottleneck is not strongly dependent on the choice of threshold, but on the fact that most intermediate genotypes have much lower fitness than the two reference genotypes.

2)The “toy” model approach and linking it with data to seem to a large degree arbitrary, “Note that other fitting functions could be used in the analysis, such as the sigmoid function” , “While the results are sensitive to the choice of φ, here for simplicity we stick to the choice made in Ref. [27], because the analysis of this section serves mostly as a motivation for the rest of the paper.” Or “To capture a few minimal ingredients inspired by the results of Sec. II A and the above discussion, we introduce a toy model featuring only global epistasis, defined as follows: … “ then other to the reader arbitrary choices follow. How is the reader supposed to believe that these choices are reasonable, supported by the data but not over fitting?

We would like to stress that we *do not* want to link the toy model to data. The data, which were previously published and analyzed, only serve as a motivation.

The main point of this paper is that a simple, stylized model of fitness landscape, featuring no network epistasis, can produce bottlenecks.

We decided to change “toy” into “stylized” model to further stress that we are not attempting any real data modeling. Our model is similar in spirit to other stylized models of evolution, such as Fisher’s geometric

model, or random fitness landscapes. These models do not attempt to model real data. They instead provide simple illustrations of what a fitness landscape may look like and can be used as “telescopes of the mind” to improve our understanding of the space of possibilities.

Because claims were made in the literature that network epistasis is necessary to have bottlenecks, we believe that our stylized model is relevant to show that this is not the case.

3)Overall, the comparison between the model and data is too qualitatively, where are proper model selection, complexity and fit error analysis? Also the authors appear to have taken some parts of the fits from another work “where we used for simplicity the same non-linear function φ−1 R (x) = φ−1 B (x) = x0.44 that was chosen in Ref. [27] to minimize the epistatic contributions.”, which further obscures what has been done here in terms of the fits.

We would like to reiterate that our aim here is not to provide the best fit to the data. Data served as an inspiration and motivation for our model, and were mostly included for pedagogical purposes.

Finding the best way to fit such kind of data on fitness landscapes has been recently the subject of debate, see e.g.

- Park et al. “The simplicity of protein sequence-function landscapes” doi:10.1038/s41467-024-51895-5
- Dupic et al. “Protein sequence landscapes are not so simple: on reference-free versus reference-based inference” doi:10.1101/2024.01.29.577800
- Park et al. “On the Analysis of Protein Genetic Architecture: Response to “Protein sequence landscapes are not so simple”” doi:10.1101/2024.09.17.613512
- Carlson et al. “Robust detection of specific epistasis using rank statistics” doi:10.1101/2025.04.08.647864

We do not want to enter into this debate. We stressed more in the revised paper that data, and their analysis, are included mostly for pedagogical purposes.

4)There are several very interesting recent developments in the field of trying to understand the structure of genotype to trait to fitness functions which are exploiting modern machine learning methods see e.g. Andreas Wagner, Genotype sampling for deep-learning assisted experimental mapping of a combinatorially complete fitness landscape, Bioinformatics, Volume 40, Issue 5, May 2024, btae317, https://doi.org/10.1093/bioinformatics/btae317

Learning the Shape of Evolutionary Landscapes: Geometric Deep Learning Reveals Hidden Structure in Phenotype-to-Fitness Maps
Manuel Razo-Mejia, Madhav Mani, Dmitri A. Petrov
doi: https://doi.org/10.1101/2025.05.07.652616

We are of course aware of efforts to model fitness landscapes using machine learning, because some of us also contributed to this effort through state of the art models such as Direct Coupling Analysis, Autoregressive Models, and Large Language Models. However, the goal here is the opposite. We are not trying to fit real data. We are trying to construct a simple, stylized model, with the goal of proving that network epistasis is not needed to have bottlenecks, contrary to some recent claims. We added a paragraph in the discussion section to specify this, and we cited the two papers suggested by the referee together with a few others.

Although in general I see value in trying to get insight with minimal modelling, I would expect a much more comprehensive and less ad-hoc/arbitrary approach to fitting the model to data to find the analysis sound and convincing.

We did not attempt any fit to real data. We only calibrated the model to produce qualitatively similar results to what was observed in the paper by Poelwijk et al.
